# Co-culture models illustrate the digestion of *Gemmata* spp. by phagocytes

**DOI:** 10.1038/s41598-018-31667-0

**Published:** 2018-09-06

**Authors:** Odilon D. Kaboré, Ahmed Loukil, Sylvain Godreuil, Michel Drancourt

**Affiliations:** 1 Aix Marseille Univ., IRD, MEPHI, IHU Méditerranée Infection, Marseille, France; 2 Université de Montpellier UMR 1058 UMR MIVEGEC, UMR IRD 224-CNRS Inserm, 1058 Montpellier, France

## Abstract

*Gemmata* spp. bacteria thrive in the same aquatic environments as free-living amoebae. DNA-based detection of *Gemmata* spp. sequences in the microbiota of the human digestive tract and blood further questioned the susceptibility of *Gemmata* spp. to phagocytes. Here, *Gemmata obscuriglobus* and *Gemmata massiliana* were co-cultured with the amoebae *Acanthamoeba polyphaga, Acanthamoeba castellanii*, *Acanthamoeba griffini* and THP-1 macrophage-like phagocytes. All experiments were performed in five independant replicates. The ratio amoeba/bacteria was 1:20 and the ratio THP-1/bacteria was 1:10. After a 2-hour co-culture, extracellular bacteria were killed by kanamycin or amikacin and eliminated. The intracellular location of *Gemmata* bacteria was specified by confocal microscopy. Microscopic enumerations and culture-based enumerations of colony-forming units were performed at T = 0, 1, 2, 3, 4, 8, 16, 24, 48 and 72 hours post-infection. Then, *Gemmata* bacteria were engulfed into the phagocytes’ cytoplasmic vacuoles, more than (98 ± 2)% of *Gemmata* bacteria, compared to controls, were destroyed by phagocytic cells after a 48-h co-culture according to microscopy and culture results, and no positive culture was observed at T = 72-hours. Under our co-culture conditions, *Gemmata* bacteria were therefore susceptible to the environmental and host phagocytes here investigated. These data suggest that these *Acanthamoeba* species and THP-1 cells cannot be used to isolate *G. massiliana* and *G. obscuriglobus* under the co-culture conditions applied in this study. Although the THP-1 response can point towards potential responses that might occur *in vivo*, these responses should first bevalidated by *in vivo* studies to draw definite conclusions.

## Introduction

Free-living amoebae (FLA) are unicellular eukaryotes commonly found in natural aquatic and soil environments^1–3^. FLA and bacteria interact in biofilms in aquatic habitats where amoebae can act as “Trojan horses” for bacterial pathogens^4–8^. Indeed, some environmental bacteria designated as Amoeba-Resisting Bacteria (ARB) have seen their mechanisms evolve to resist amoebae and use amoebae as replicative niches^4–6^. In 1980, Robowtham demonstrated that the bacterial pathogen *Legionella pneumophila* was a prototype ARB^4^ and several ARB have been further discovered, including, among others, *Coxiella burnetii*^5^, several mycobacteria^6–12^ and *Parachlamydia acanthamoebae*, a member of the so-called *Planctomycetes*, *Verrucomicrobia*, *Chlamydiae* (PVC) superphylum^13,14^.

*Planctomycetes* are members of terrestrial and aquatic microbial communities^15–20^. These organisms have been regularly isolated from various habitats including soil, freshwater lakes, seawater, brackish water lagoon, wastewater habitats and river biofilms^15–23^. In humans also, the DNA of *Planctomycetes* has been detected in the gut microbiota^24^ where *Akkermancia mucophila* has been isolated^25,26^. Only two representative organisms of the genus *Gemmata* have been cultured, including *Gemmata obscuriglobus*, first isolated in 1984 from a freshwater dam in Australia^17^, and *Gemmata massiliana*, that we recently isolated from a hospital water system in close proximity to patients^18^. Therefore, patients may be exposed to these microorganisms when drinking freshwater or water used for health care such as hydrotherapy baths. Accordingly, *Gemmata* DNA sequences have been detected in the human digestive microbiota^24^ as well as in the blood collected from two aplastic leukemic febrile patients with neutropenia^27^. *Gemmata* spp. bacteria are multimultidrug-resistant *Planctomycetes*^28^ and may be opportunistic pathogens in selected patient populations^27^.

In the environment, *Gemmata* may be in close contact with FLA, which normally feed on bacteria by phagocytosis. Amoeba co-culture has been used to isolate some fastidious microorganisms such as members of *Legionella*^4^ and waterborne mycobacteria including *Mycobacterium massiliense*^12^. In addition, amoebae may be used as model organisms to investigate the relationship between bacteria and phagocytes, including macrophages^29^. Some studies reported the relationship between planctomycetes and aquatic microbial communities such as sponges^23,30^ and macroalgae^31–33^, but the interactions between amoebae and *Gemmata* bacteria have not been specifically explored.

Here, we investigated the relationship between phagocytic amoebae and THP-1 cells with the two cultured *Gemmata* species. After preliminary experiments ensuring the viability of both *Gemmata* species in the medium used to culture amoebae and in the medium used to culture macrophages, both *Gemmata* species were exposed to amoebae and macrophages and the intracellular uptake and survival of bacteria were observed by optic and confocal microscopy, and colony-forming unit measurement.

## Results

### Preliminary experiments

Prior to co-culture experiments, we assessed the survival of both *Gemmata* species in the media used to culture amoebae and THP-1 cells. As for the survival of 2.10^7^ *Gemmata*/mL in PAS (Page’s Amoebal Saline) and RPMI 1640 (Roswell Park Memorial Institute), the number of medium colony-forming-units (CFU)/mL was (1.84 ± 0.28) × 10^7^ and (1.92 ± 0.13) × 10^7^ for *G. obscuriglobus* and *G. massiliana*, respectively, whereas it was (2.01 ± 0.31) × 10^7^ for *G. obscuriglobus* and (2.03 ± 0.17) × 10^7^ CFU/mL for *G. massiliana*. Likewise, the number of *G. obscuriglobus* CFU/mL obtained with the suspension contained in PAS was (1.78 ± 0.98) × 10^7^ and (1.87 ± 0.24) × 10^7^ for *G. massiliana* (P > 0.05) at day 3. Co-culture experiments were performed including an optimized (after preliminary experiments) two-hour inoculation of phagocytes with *Gemmata* bacteria to allow for a greater duration of bacterial uptake. Extra-phagocyte bacteria were killed by antibiotic treatment with 82 ± 5% and 92 ± 2% of *G. obscuriglobus* death in PAS and RPMI medium, respectively; and 86 ± 7% and 89 ± 4% of *G. massiliana* death in PAS and RPMI medium, respectively. Furthermore, inoculated phagocytes were washed three times and the third washing was microscopically observed and cultured on solid agar. Microscopic observation showed 24 ± 8/µL phagocytes and 31 ± 13/µL *Gemmata* bacteria. Culture remained sterile except for seven *G. obscuriglobus* colonies observed in one of the replicates of the *G. obscuriglobus-A. castellanii* co-culture. Light microscopy of co-culture at T = 0 (“time of beginning“after the third wash) showed intra-phagocyte *Gemmata* spp. and a few extracellular bacteria. Heat-shock procedure lysed 96–100% amoebae and 98–100% THP1-cells (trypan blue staining). The effects of thermal shock on *Gemmata* spp. viability included a loss rate of (2.48 ± 1.02) % (1.95 ± 0.09) × 10^7^ CFU/mL remaining alive) for *G. obscuriglobus* and (7.15 ± 2.24) % (1.88 ± 0.18) × 10^7^ CFU/mL remaining alive) for *G. massiliana*, using an initial suspension of 2.10^7^ CFU/mL (P > 0.05). The sterility of the third washing and of *G. obscuriglobus* and *G. masiliana*-inoculated amoebae as well as that of the THP-1 cells not submitted to thermal shock confirm that further culture observations from the thermal shock lysates indeed derive from intracellular bacteria.

### *Gemmata* spp.-amoebae co-culture

**Figure 1:**
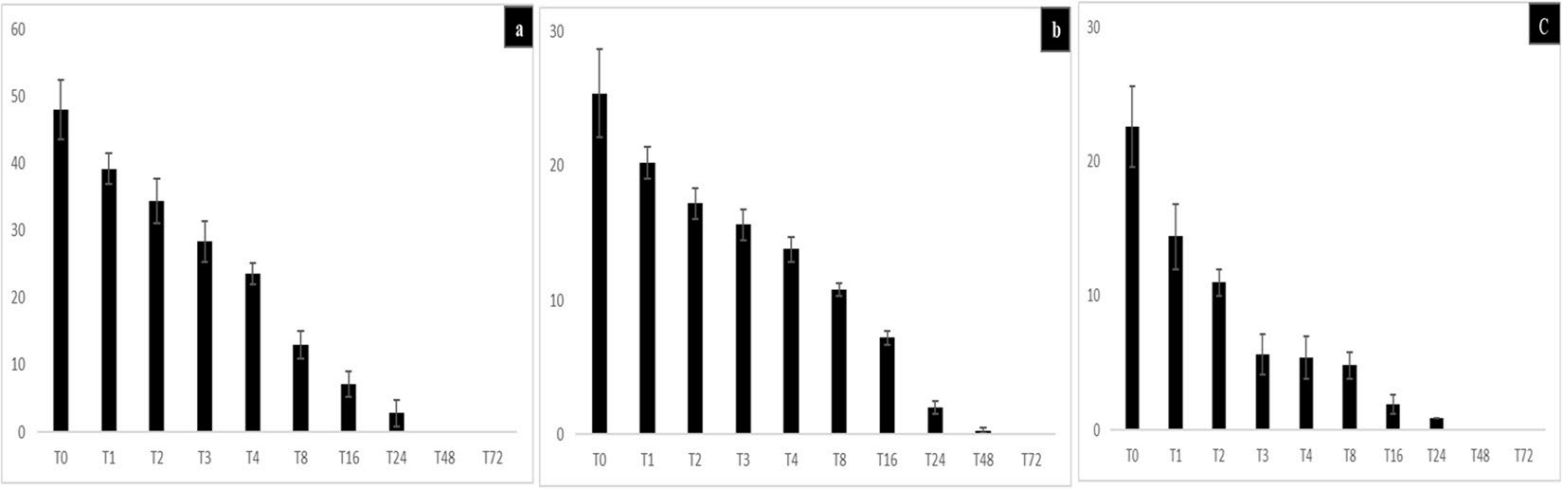
(**a**–**c**) Percentage of intracellular *G. obscuriglobus* bacteria observed by fluorescence microscopy after acridine orange staining in trophozoites of *A. polyphaga* (**a**), *A. castellanii* (**b**) and *A. griffini* (**c**). X axis figures time (hours) of coculture, Y axis figures the percentage of intracellular bacteria per 100-amoebae counted, standard errors are figured by error bars.

**Figure 2:**
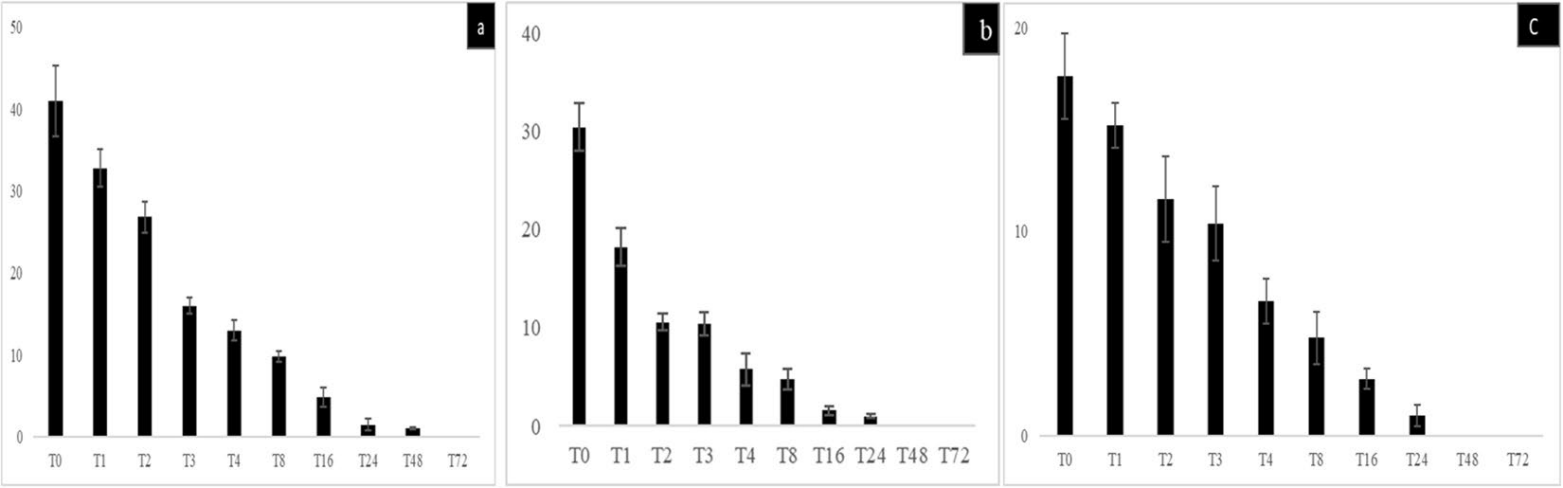
(**a**–**c**) Percentage of intracellular *G. massiliana* bacteria observed by fluorescence microscopy after acridine orange staining in trophozoites of *A. polyphaga* (**a**), *A. castellanii* (**b**) and *A. griffini* (**c**). X axis figures time (hours) of co-culture, Y axis figures the percentage of intracellular bacteria per 100-amoebae counted, standard errors are figured by error bars.

**Figure 3:**
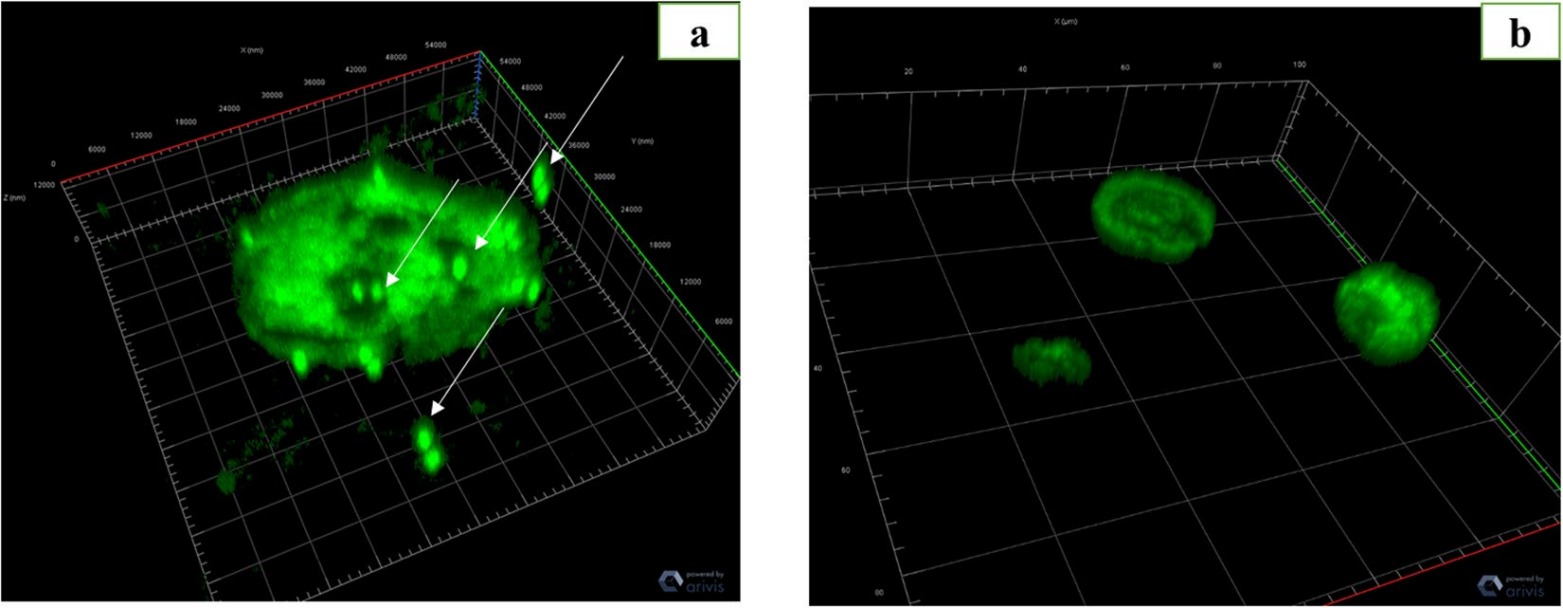
(**a,b**) Confocal microscopy after acridine orange staining showing *G. massiliana* (white arrows) within and outside of *A. griffini* trophozoite at T0 h (**a**) and negative control (uninfected *A. griffini* (**b**) at T0 h. Magnification 63X/1.4 oil objective, 3D reconstruction after z-stack in Zeiss LSM 800 confocal microscopy.

**Figure 4:**
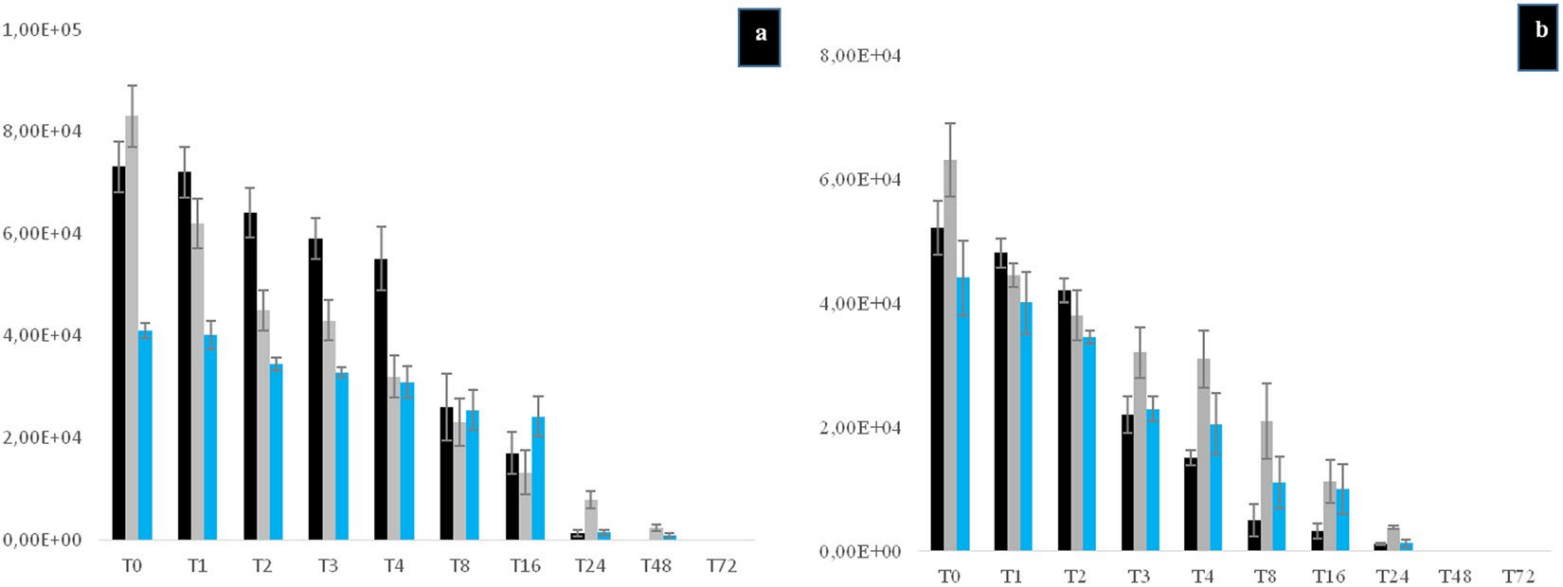
(**a,b**) Cultures of lysate performed after the thermal shock of *G. obscuriglobus* (**a**) and *G. massiliana* (**b**) with *A. polyphaga* trophozoites (blackbars), *A. castellanii* (gray bars) and *A. griffini* (blue bars). X axis figures time (hours) of co-culture, Y axis figures the number of CFUs, standard errors are figured by error bars.

The reproducibility of the results here reported was ensured by five successive and independant experiments in which all the non-infected, negative-control amoebae remained *Gemmata*-free. In these co-culture experiments, the number of non-inoculated, negative-control amoebae and *Gemmata* spp.-containing amoebae did not change significantly over time (p < 0.05). Accordingly, no amoebal lysis and no cysts were observed by microscopy after day 3 of the experiment in negative controls and *Gemmata* spp. -containing amoebae. After a 2-hour co-culture and antibiotic treatment followed by series of washes at T0, the percentage of *G. obscuriglobus-*containing amoeba trophozoites was of (48.2 ± 4.5)% for *A. polyphaga* **(**Fig. 1a), (25.4 ± 3.4)% for *A. castellanii* **(**Fig. 1b) and (22.6 ± 3.2)% for *A. griffini* **(**Fig. 1c) (P > 0.05) and the percentage of *G. massiliana-*containing amoeba trophozoites was of (41 ± 4.4)% for *A. polyphaga* (Fig. 2a), (30.4 ± 2.4)% for *A. castellanii* **(**Fig. 2b) and (17.6 ± 2.1)% for *A. griffini* (P > 0.05) **(**Fig. 2c**)**. At T = 0, *Gemmata* spp.-containing amoebae then contained between 1 and 7 *G. obscuriglobus* bacteria and between 1 and 5 *G. massiliana* bacteria per amoebal trophozoite. Some bacteria were located into vacuoles, as confirmed by confocal microscopy. Three-dimensional (3D) reconstruction after z-stack acquisition using confocal laser microscopy (Zeiss LSM 800) showed the presence of internalized green fluorescent bacteria within vacuoles inside infected amoebae. However, no bacteria were observed within uninoculated amoebae. In *A. griffini*, the amoeba contained 3.67 ± 2.51 *G. massiliana* and 4.26 ± 2.77 *G. obscuriglobus*, including 2 ± 1 *Gemmata* spp. located in vacuoles (Fig. 3a), whereas non-inoculated control amoebae did not exhibit bacteria or vacuoles (Fig. 3b). In successive time-point observations, the number of intra-amoebal bacteria decreased, resulting in 98 ± 2% of *Gemmata* spp. bacteria being digested by amoebae after a 48-h co-culture. In parallel, the number of *Gemmata* spp. CFUs performed with the lysate after thermal shock decreased significantly (P < 0.0001) from T = 0 -hour to T = 48 -hours. Indeed, in *G. massiliana*-A*. polyphaga* co-culture, the number of CFUs decreased from (5.21 ± 0.29) × 10^4^ at T = 0 -hour to (0.32 ± 0.12) × 10^4^ at T = 16 -hours, (0.11 ± 0.02) × 10^4^ at T = 24 (P < 0.0001) and limit of detection at T = 48 -hours for. For *G. obscuriglobus-A. polyphaga* co-culture, CFUs decreased from (7.32 ± 0.50) × 10^4^ at T = 0 -hour to (1.70 ± 0.40) × 10^4^ at T = 16 -hours, (0.13 ± 0.07) × 10^4^ at T = 24 (P < 0.0001) and limit of detection at T = 48 hours. In *A.castellanii-G.massiliana* co-culture, the number of CFUs decreased from (6.30 ± 0.30) × 10^4^ at T = 0 -hour to (1.10 ± 0.26) × 10^4^ at T = 16 hours, (0.38 ± 0.03) × 10^4^ at T = 24 (P < 0.0001) and limit of detection at T = 48 hours. In *G. obscuriglobus-A.castellanii* co-culture, CFUs decreased from (8.30 ± 0.61) × 10^4^ at T = 0 -hour to (1.32. ± 0.43) × 10^4^ at T = 16 hours, (0.73 ± 0.17) × 10^4^ at T = 24 and (0.16 ± 0.06) × 10^4^ at T = 48 hours (P < 0.0001). In *A. griffini* co-culture, CFUs decreased from (4.30 ± 0.27) × 10^4^ for *G. massiliana* at T = 0 -hour to (0.90 ± 0.34) × 10^4^ at T = 16 -hours, (0.20 ± 0.02) × 10^4^ at T = 24 (P < 0.001) and limit of detection at T = 48 hours and for *G. obscuriglobus* from (4.10 ± 0.15) × 10^4^ at T = 0 -hour to (2.42 ± 0.39) × 10^4^ at T = 16 -hours, (0.15 ± 0.05) × 10^4^ at T = 24 and (0.09 ± 0.05) × 10^4^ at T = 48 hours (P < 0.0001). No positive culture was observed at T = 72 -hours for the two *Gemmata* species co-cultured with any amoebal lysate (Figs. 4a, 4b). Compared to controls not submitted to thermal shock, the amoebae did not lead phagocyted *Gemmata* bacteria out of their cytosol, since no growth was observed. More than (98 ± 2%) of the *Gemmata* spp. organisms were internalized and destroyed by amoeba after a 48-h co-culture according to microscopic results, and no positive culture was observed at T = 72 -hours. Overall, no replication occured under the experimental conditions here reported. Compared to non-inoculated amoebae controls, almost all amoebae possessed many vacuoles without bacteria at T = 72 -hours. *Gemmata* spp. bacteria became indetectable by microscopic examination and by subculturing on agar plates at day 3. No discrepancy was observed between microscopy and culture results.

### *Gemmata* spp.-THP-1 cells co-culture

**Figure 5:**
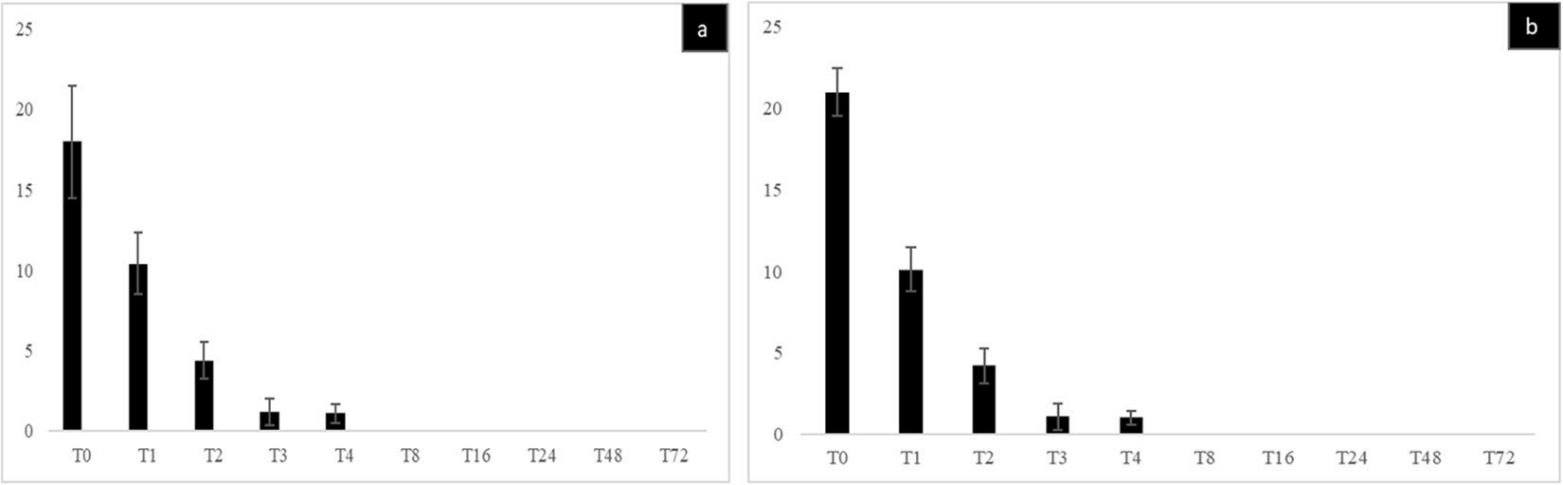
(**a,b)** Intracellular survival of *G. obscuriglobus* (**a**) and *G. massiliana* (**b**) within THP-1 cells. X axis figures time (hours) of co-culture and Y axis figures the percentage of infected THP-1 cells, standard errors are represented by error bars.

**Figure 6:**
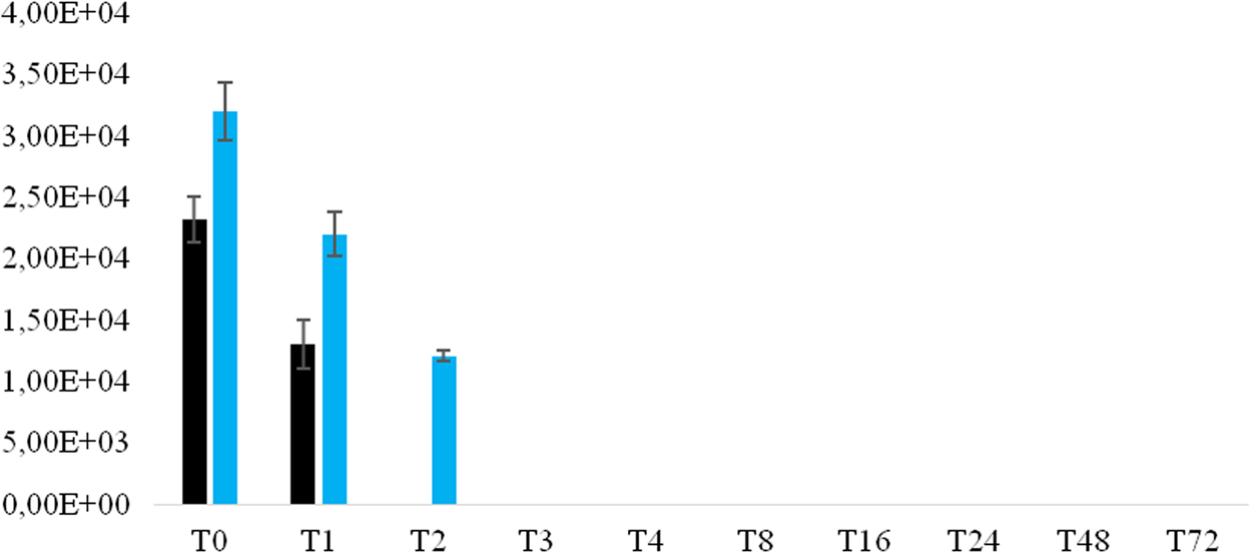
Cultures of lysate of THP-1 cells infected with *G. obscuriglobus* (black bars) or *G. massiliana* (blue bars). X axis figures time (hours) of co-culture, Y axis figures the number of CFUs, standard errors are figured by error bars.

The reproducibility of the results here reported was ensured by five successive and independant experiments in which all the non-infected, negative-control THP-1 cells remained *Gemmata*-free. In a first step, we observed that *Gemmata* spp. bacteria survived for 72 hours in RPMI. Then co-culture experiments and microscopic examination of THP-1 cells after trypan blue staining indicated no significant difference in the viability of THP-1 cells over time, whether they be *Gemmata* spp. -containing THP-1 or not. THP-1 cells viability was of (95.6 ± 2.7) % (non- inoculated) and (96 ± 2.1) % (inoculated THP-1 cells) at day 1, *versus* (93.2 ± 2.4) % (non- inoculated) and (91 ± 3.2) % (inoculated THP-1cells) at day 3 post-inoculation (P > 0.05). After a 2 -hour co-culture and antibiotic treatment followed by series of washes at T0, (18 ± 4) % of THP-1 cells were found to be *G. obscuriglobus*.-containing THP-1 and (21 ± 2) % were found to be *G. massiliana*-containing THP-1 (p > 0.05). Then, at T = 1, T2, T3, T4 and T8 until 72 -hours post-culture, the number of *G. obscuriglobus*-containing THP-1 cells decreased from (10.4 ± 2)%, (4.4 ± 2)%, (1.2 ± 1)% and (1.1 ± 1) % to limit of detection at T = 8 until day 3 of co-culture, respectively (Fig. 5a). For *G. massiliana*, At T = 1, T2, T3, T4 and T8 until 72 hours post-culture, the number of *G. massiliana -*containing THP-1 cells decreased from (10.1 ± 1.4) %, (4.2 ± 1.4) %, (1.1 ± 1) %, and (1.0 ± 0.4) % to limit of detection at T = 8 until day 3 of co-culture, respectively, as showed by Fig. 5b. The number of *Gemmata* organisms per THP-1 cell varied from 1 to 2 whereas negative controls remained *Gemmata*–free. The number of non-inoculated (negative-control) and *Gemmata* spp.-containing THP-1 cells did not change significantly during the experiment. Likewise, the number of *G.massiliana* CFUs performed with culture lysate decreased from (3.2. ± 2.4) × 10^4^ at T = 0 -hour, to (2.2 ± 1.8) × 10^4^ at T = 1 to (1.2 ± 0.4) × 10^4^ at T = 2 -hours (P < 0.001), tolimit of detection from T = 3 to T = 72 hours. For *G. obscuriglobus*, the number of CFUs decreased from (2.3. ± 1.8) × 10^4^ at T = 0 -hour to (1.3 ± 0.2) × 10^4^ at T = 1 and limit of detection from T = 2 to T = 72 hours (Fig. 6). THP-1 cells did not allow for the multiplication of *Gemmata* organisms that became indetectable by means of microscopic examination of phagocytic cells or by subculturing on agar plates at day 3 of co-culture.

## Discussion

Amoebae and THP-1 cells are phagocytic cells which were previously used to probe microorganism-phagocyte interactions^34–39^. In the present study, we investigated such interactions between only two culturable representatives of the bacterial genus *Gemmata*, i.e. *G. obscuriglobus* and *G. massiliana*. In these series of experiments, culture of the washing product and controls not submitted to thermal shock action of amoebae and THP-1 cells inoculated with *G. obscuriglobus* or *G. masiliana* remained sterile, indicating that no extracellular bacteria remained alive or escaped from the cytosol of phagocytic cells, and that all further observations in culture of lysate obtained after thermal shock derived from intracellular bacteria. Also, the data presented in this study were interpreted as authentic because negative controls remained negative in each experimental step. At last, the observations were reproduced five times.

In every co-culture experiment, amoebae and THP-1 cells were found to be bactericidal against the two investigated *Gemmata* spp. The fact that results obtained with amoebae mirror results obtained with THP-1 cells is not surprising, as demonstrated previously with the similar actions of these phagocytic cells in numerous bacteria such as *Legionnela*, many mycobacteria and *Chlamydia*, which can resist both amoebae and macrophages^4,6,13^.

Results obtained with the amoebae here investigated suggest that these unicellular eukaryotes are unlikely hosting *Gemmata* species in the aquatic environments where they could thrive together. *Gemmata* are mainly found in soil and aquatic environments along with FLA but have also been found in the human gut microbiota, in the blood of immune-compromised aplastic leukemic patients^17,18,24,26^ and hospital water networks in close proximity to patients^18^. These recent observations in our laboratory led us to conduct also *Gemmata* spp-TPH-1 cells co-culture to obtain insights into the link between phagocytose and monocyte-macrophage cells in human infection. Using a low (1:10) multiplicity of infection (M.O.I) with THP-1 and hight M.O.I (1:20) with amoebae, we obtained reproducible results in five independant experiments. We observed that *Gemmata* spp. were rapidly internalized and digested by amoebae and THP-1.

This high susceptibility of *Gemmata* spp. to bactericidal phagocytosis contrasted with the fact that *Gemmata* spp. are multidrug-resistant bacteria^28^ and have a high ability to adapt to harsh environments. Moreover, these bacteria may thrive in the human body^24,27^. However, under the co-culture conditions of our experiments, *Gemmata* bacteria did not resist to amoebae and THP-1 bactericidal action despite their panoply of attack and defense mechanisms^40^. The rapid and significant phagocytosis of *Gemmata* bacteria could be explained by the presence of holdfasts of glycoproteic nature on their outer membrane^16,18^ which could facilitate adhesion to phagocytic cell receptors and increase cell-to-cell contact and rapid internalization. Consequently, *Gemmata* spp. life cycle requires an attached state. Their proliferation starts when they attach through their holdfast to a bracket. Then, flagellated budding new cells are formed which move freely in the water until developing their holdfast and returning to the attached form^41^. FLA and THP-1 cells do not offer these survival and proliferation conditions compared to macroalgae, marine sponges and crustaceans, which are closely related to planctomycetes in aquatic environments and biofilms^31^.

In conclusion, the data here reported show that coculturing the two *Gemmata* species under investigation with *Acanthamoeba* amoebae results in *Gemmata* death. *Acanthamoeba* ameobae are unlikely hosting *Gemmata* spp. in the environment. Moreover, these amoebae could not be used for the tentative isolation of *Gemmata* bacteria in the laboratory under the co-culture conditions tested in this study. Moreover, the interactions between THP-1 cells and *Gemmata* spp. could provide insight into the action of the monocytes–macrophages against *Gemmata* spp. cells during the colonization and infection. Though unlikely, patients with compromised macrophage activities, such as aplastic patients, could be infected by opportunistic *Gemmata* spp. Although the THP-1 response can hint to potential responses that might occur *in vivo*, these reponses should first be validated by *in vivo* studies before drawing more definite conclusions.

## Materials and Methods

### Preliminary experiments

The methodological procedures here presented have been designed and standardized after a series of preliminary studies. Preliminary experiments were used to define the phagocyte/bacteria ratio; the duration of co-culture of 6 h, 4 h, 3 h and 2 h before T0; antibiotic treatment conditions (concentration and duration of treatment) to kill rapidly extracellular bacteria, and the effectiveness of the wash in removing extra-cellular bacteria (later controlled by microscopy and culture). Also, different types of staining (Giemsa, Gimenez, Hemacolor, acridin orange) were tested to choose the most appropriate staining, and the thermal shock procedure was evaluated and standardized before the experiments. Then, the protocol was successively applied with each amoeba species and to THP-1 cells.

### Bacterial strains and culture conditions

*G. obscuriglobus* DSM 5831^T^ and *G. massiliana* DSM 26013^T^ (CSUR P189^T^) were obtained from the Collection de Souches de l’Unité des Rickettsies, Marseille, France, and the German Collection of Microorganisms and Cell Cultures (Braunschweig, Germany). Bacteria of both species were sub-cultured on Caulobacter medium DSMZ 595 supplemented by 5% *Escherichia coli* filtrate or Staley’s maintenance medium DSMZ 629 prepared as described on the website (http: //www.dsmz.de). Bacteria were grown on these solid media incubated aerobically at 30 °C for 7 to 14 days. Identification of colonies was ensured by matrix-assisted laser desorption/ionization time-of-flight mass spectrometry (MALDI-TOF-MS) analysis as previously described^42^. Prior to co-culture, colonies were harvested in a 15-mL tube containing 5 mL of sterile phosphate-buffered saline (PBS), the tube was rigorously vortexed and the suspension was passed three times through a 29-Gauge needle in order to separate aggregates. The inoculum was adjusted at 2 × 10^7^ cells colony-forming units (CFU)/mL after calibration by Kovas slide 10 (Hycor Biomedical, Indianapolis, IN, USA) for co-culture. In parallel, this inoculum was maintained in RPMI 1640 and PAS for three days to check *Gemmata* spp viability after one to three days and compared with Staley’s liquid medium as reference. Culture-based microbial enumeration on Staley’s solid agar has been performed in order to assess bacterial survival in these co-culture liquid media.

### Amoebae and culture conditions

*Acanthamoeba polyphaga* (strain Linc AP1), *Acanthamoeba castellanii* (strain ATCC 30234) and *Acanthamoeba griffin* (strain ATCC 50702) were cultured independently in axenically Peptone Yeast-extract Glucose (PYG) medium placed in 75-cm^2^ culture flasks. The flasks were then incubated for 48 hours at 30 °C for *A. polyphaga* and 28 °C for *A. griffini* and *A. castellanii*. Trophozoites were suspended by tapping the flasks, centrifuged in 50-mL tubes and the pellet was suspended in Page’s amoebal saline (PAS). Then, the amoebal cells were adjusted at 10^5^ cells/mL using Kovas slide 10 and we checked their viability using trypan blue staining before co-cuture. Amoeba viability and growth were assessed at the end of co-culture (day 3) using trypan blue staining.

### Human monocytic THP-1 cell lines and culture conditions

THP-1 cells were retreived from the blood of a patient with acute monocytic leukemia. Since their establishment in 1980^43^, THP-1 cells have become one of most widely used cell lines to investigate the function and regulation of monocytes and macrophages in the cardiovascular system. After exposure to phorbol-12-myristate-13-acetate (PMA, also known as TPA, 12-O-tetradecanoylphorbol-13-acetate), nearly all the THP-1 cells started to transform into macrophages^44^. In this study, unstimulated THP-1 cells were preferred over macrophages (present in the tissues) in order to mimic the behavior of peripheral blood monocytes cells. Indeed, *Gemmata* DNA has been previously detected in the blood collected from two aplastic leukemic febrile patients but not in tissues. THP-1 cells were kindly provided by Dr. E. GHIGO, IHU Méditerranée Infection, Marseille, France. Cells were grown in RPMI 1640 (Gibco™, Eggenstein, Germany) tissue culture medium supplemented with 2% glutamine and 10% heat-inactivated fetal bovine serum in 75-cm^2^ tissue culture flasks, incubated at 37 °C in 5% CO_2_. The culture medium was refreshed every 3 days. Prior to co-culture, cells were harvested and washed thoroughly twice with PBS and adjusted at 2 × 10^6^ cells/mL using Kovas slide 10. We checked their viability using trypan blue staining before co-cuture. The viability and growth were assessed at the end of co-culture (day 3).

### Amoeba-*Gemmata* species co-culture conditions

All experiments were performed in five independant replicates during 72 hours in 12-well tissue culture plates (Becton Dickinson, Le Pont-de-Claix, France) and repeated five times for ensuring reproducibility. Each species of amoeba was co-cultured with each *Gemmata* species. Each plate contained six wells with amoebae co-cultured with each *Gemmata* species and six wells with amoebae as negative control wells (bacteria-free). More precisely, 1.8 mL of the amoebae-containing suspension was pipetted in each well of a 12 well-plate, 200 μL of *Gemmata* suspension at 2 × 10^7^ CFU/mL in PBS (ratio amoebae/bacteria was 1:20) was added in challenged wells and 200 μL of PBS in control wells. Plates were incubated at 30 °C for two hours. After a 2 -hour co-culture, in order to eliminate extracellular bacteria, the supernatant was removed, the amoeba monolayer was washed twice and two milliliters of modified PAS containing 150 g/L kanamycin was added in each well, including negative control wells. After a 30-min incubation period with the antibiotic, the amoeba monolayer was rinsed twice with PBS to eliminate the extracellular bacteria, this operation was repeated once with a 30-min incubation period and then rinsed twice to obtain a third washing. Antibiotic treatment protocol in 2 × 30 min was chosen to combine inactivation and physical removal of extracellular bacteria to have low extracellular bacteria in the last rinse product. Antibiotic inactivation tests of *Gemmata* spp were performed in PAS in the presence or absence of phagocytic cells for the interpretation of culture results. Finally, after a series of antibiotic rinsing, the amoeba monolayer was covered with two mL of PAS in each well and the plates were incubated at 30 °C for *A. polyphaga* and 28 °C for *A. griffini* and *A.castellanii* for the rest of the experiment. Negative controls (uninfected) of each amoeba were cultured separately in PAS medium as described above.

### THP-1 cells*-Gemmata* spp.co-culture conditions

Co-culture was performed in RPMI 1640 medium without fœtal bovine serum and glutamine in order to deplete the stored nutrients and slow down the growth of THP-1 cells. The experiments were performed in five independent replicates during 72 hours in 12-well tissue culture plates, each containing independently *Gemmata* species, as previously described for amoebae. Each plate contained six wells with THP-1 cells co-cultured with each *Gemmata* species and six wells with THP-1 cells as negative control wells (bacteria-free). More specifically, 1.8 mL of 2.10^6^ cells/mL THP-1 cells (calibrated as described above) suspension was pipetted in each well of a 12-well plate, 200 μL of *Gemmata* suspension at 2 × 10^7^ CFU/mL (ratio THP-1 cells/bacteria was 1:10) was added in challenged wells and 200 μL of PBS in control wells. Plates were incubated at 37 °C with 5% CO_2_ for two hours. After a 2 -hour co-culture, in order to eliminate extracellular bacteria, the supernatant was removed, THP-1 cells were washed with PBS and two mL of RPMI containing 150 g/L of amikacin was added in all wells, including negative control wells for 30 min. After a 30-min incubation period at 37 °C under 5% CO_2_ with the antibiotic, the cells were rinsed to eliminate the extracellular bacteria, then antibiotic treatment was repeated once with a 30-min incubation period and then rinsed twice to obtain the last rinse called washing product. 100 μL of this washing product was cultured in Staley’s medium to ensure the absence of viable extracellular bacteria. Microscopic controls (using Kova slide) at the fresh state of the washing product were performed, as described above, before being plated on solid agar. Finally, THP-1cells were covered with 2-mL of RPMI in each well and the preparations were incubated at 37 °C in the presence of 5% CO_2_ for 72 hours_._ Uninfected THP-1 cells were cultured in the same conditions as negative controls.

### Microscopy and culture conditions of lysate obtained after thermal shock

After a 2 -hour co-culture duration, antibiotic treatment and a series of rinsing, microscopic enumerations and culture-based enumerations of colony-forming units were performed at T = 0 (time beginning after the last rinse of antibiotic), 1, 2, 3, 4, (close kinetic counting to see more intracellular bacteria phagocyted at the beginning) and T 8, 16, 24, 48, 72 -hour post-inoculation (procedure standardized after preliminary sudies).

### Microscopy

The phagocyte cells and the supernatant were removed from each well, 200-µL volume was used to prepare a Cytospin (smears) centrifuged at 44 g during 5 mins for microscopy analysis. Smears were prefixed with 90° ethanol. Amoeba-*Gemmata* spp coculture smears were examined by fluorescence microscopy after acridine orange staining as described on the website http://www.memobio.fr/html/bact/ba_te_acr.html. In order to precise the intracellular location of bacteria, smears were observed under a Zeiss LSM 800 confocal microscope using a 488 nm excitation laser (Carl Zeiss S.A.S., Marly-le-Roi, France). The 63X/1.4NA oil immersion objective was used for image acquisition. 3D reconstruction was performed using Zen software (Zeiss) from a z-stack acquisition of 10 images with a z-spacing of 1.2 µm. Images were post-processed using ImageJ software by adjusting contrast and brightness. For THP-1-*Gemmata* co-culture, slides were stained by the Gimenez method and obseved by light microscopy. Microscopic results were expressed by counting the number of intracellular bacteria per 100-amoeba or THP-1 counted. Means and standard errors of five independent experiments have been calculated using Excel 2013 software. The phagocytic cells and the supernatant were removed from each well, a 200-µL volume was used to prepare a Cytospin (smears) centrifuged at 44 g during 5 mins for microscopy analysis. Smears were prefixed with 90° Ethanol. Amoeba-*Gemmata* spp co-culture smears were examined by fluorescence microscopy after acridine orange staining as described on the website http://www.memobio.fr/html/bact/ba_te_acr.html. In order to observe intrabacteria localization in vacuoles or in the cytoplasm, smears were observed under a Zeiss LSM 800 confocal microscope using a 488 nm excitation laser (Carl Zeiss S.A.S., Marly-le-Roi, France). The 63X/1.4NA oil immersion objective was used for image acquisition. 3D reconstruction was performed using Zen software (Zeiss) from a z-stack acquisition of 10 images with a z-spacing of 1.2 µm. Images were post-processed using ImageJ software by adjusting contrast and brightness. For THP-1-*Gemmata* co-culture, slides were stained by the Gimenez method and obseved by light microscopy. Microscopic results were expressed by counting the number of intracellular bacteria and extracellular bacteria (remaining bacteria not removed by last rinsing) per 100-amoebae or THP-1 counted. Means and standard errors of five independant replicates have been calculated using Excel 2013 software.

### Culture conditions of lysate obtained after thermal shock

To determine the viability of intra-phagocyte bacteria, 200 μL of the phagocytic monolayer and the supernatant were removed from the bottom of wells and transferred in 1.5-mL polypropylene tubes. The preparation was submitted to thermal shock. It was frozen in liquid nitrogen at −196 °C, submitted to direct defrosting in a water bath at 40 °C for two minutes and then vortexed for 30 seconds. This operation was repeated once to ensure more lysis of host cells (Phagocytic lysis was checked using Trypan blue staining). A 100-μL volume of the lysate and 100 μL of a one-fold dilution in PBS were cultured for two weeks in Staley’s medium and incubated at 30 °C to quantify CFU. A 100-μL volume of culture product not subjected to the thermal shock action was cultured in parallel for control. Colonies were counted after 2 weeks of growth at 30 °C. Futhermore, in order to assess the impact of thermal shock on the viability of *Gemmata* spp. bacteria, the *Gemmata* inoculum at 2.10^7^ bacteria/mL was subjected to the thermal shock procedure and then plated on staley’s agar medium to check its viability. Data are expressed as CFU per milliliter. Means and standard errors have been calculated using Excel 2013 software.
